# Supplementary figures and images for: Heterotopic Cesarean Scar Pregnancy: A Systematic Review of Diagnosis, Management and Prognosis
Source: Diagnostics (Basel). 2025 Sep 18;15(18):2373. doi: 10.3390/diagnostics15182373 (PMC12468486; doi:10.3390/diagnostics15182373)

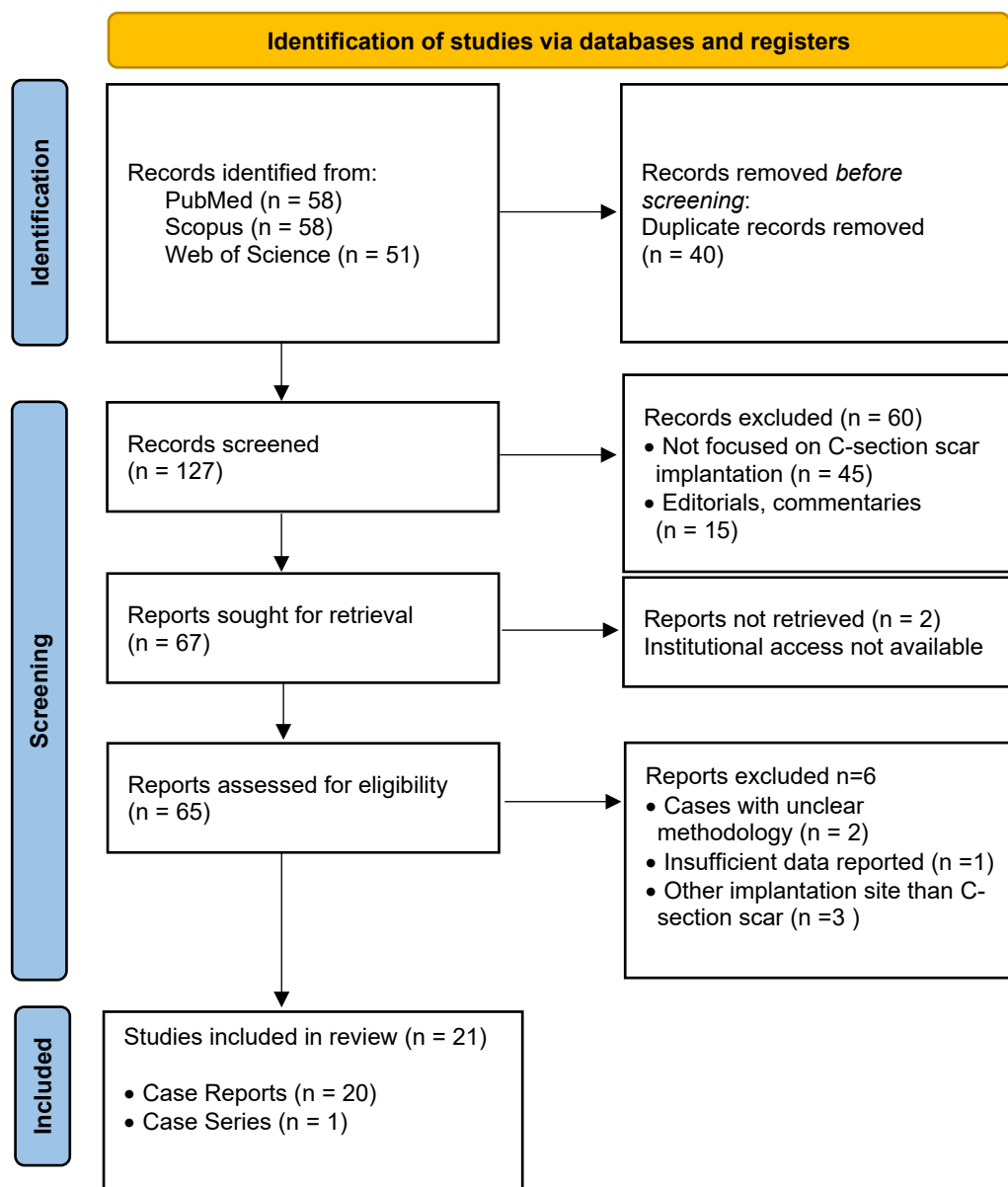

Supplement: Supplementary file 1 [file diagnostics-15-02373-s001.zip › diagnostics-3859521-Supplementary Material 2 - PRISMA 2020 flow diagram.pdf]
